# 520 Use of Phenobarbital for Alcohol Withdrawal in Small Burn Patients: Evaluation of a Pilot Protocol

**DOI:** 10.1093/jbcr/irad045.117

**Published:** 2023-05-15

**Authors:** Zachary Drabick, Amalia Cochran, Ian Driscoll

**Affiliations:** UF Health Shands Burn Center, High Springs, Florida; UF Health Shands Burn Center, Gainesville, Florida; UF Health Shands Burn Center, Gainesville, Florida; UF Health Shands Burn Center, High Springs, Florida; UF Health Shands Burn Center, Gainesville, Florida; UF Health Shands Burn Center, Gainesville, Florida; UF Health Shands Burn Center, High Springs, Florida; UF Health Shands Burn Center, Gainesville, Florida; UF Health Shands Burn Center, Gainesville, Florida

## Abstract

**Introduction:**

Phenobarbital is a long-acting barbiturate that has primarily been used an antiepileptic in clinical practice. However its use as a sedative has seen a resurgence recently especially for the prevention and/or treatment of alcohol withdrawal. It has several characteristics that make it appealing for this indication, including its long half-life and its distinct binding to GABA_A _receptors that, unlike benzodiazepines, does not depend on endogenous GABA to produce its effects. Several recent studies have evaluated its use for the treatment of alcohol withdrawal both inside and outside of the ICU setting, however reports of its use in patients with burn injury are sparse. A pilot treatment protocol utilizing phenobarbital for patients with less than 20% TBSA burns at high risk of alcohol withdrawal was formulated and put into practice at our center in January of 2022. We sought to evaluate the safety and efficacy of this protocol for quality improvement.

**Methods:**

This was a retrospective chart review of patients admitted from January to August 2022 with less than 20% TBSA burns who received treatment with phenobarbital for alcohol withdrawal at a major burn center in North Central Florida. There were no exclusion criteria. This project was registered as a Quality Improvement Project with the institution.

**Results:**

Ten patients were included in the evaluation. Median age was 63 yo, 90% were male, and 70% were Caucasian. Median % TBSA burn was 6.5% (range 0.5-12%) and median length of stay was 16 days (range 9-50 days). Most patients were injured by Fire/Flame (80%). Most patients had a history of binge drinking (70%), and most reported drinking at least 3-4 alcoholic drinks 4 or more times per week (70%). Of those that had a detectable blood alcohol concentration on admission, the median was 163 mg/dL (range 44-325 mcg/dL). Over half of the patients (60%) received an IV bolus (either 5 or 10 mg/kg based on ideal body weight) of phenobarbital followed by an oral taper, with the remaining 40% having received an oral taper without a bolus dose. Median RASS prior to initiation of the protocol was +1, and the majority of patients remained RASS +1 or less throughout the duration of the protocol (70%). No rescue benzodiazepines were needed, no seizures were identified, and no intubations occurred while patients were on the protocol.

**Conclusions:**

The phenobarbital protocol was well tolerated and controlled agitation in the majority of the patients who received it. No obvious safety concerns were identified during this review. Although this was a small sample size, these results are encouraging and support continued use of phenobarbital in preventing and controlling symptoms of alcohol withdrawal in patients with less than 20% TBSA burns.

**Applicability of Research to Practice:**

Phenobarbital may be a safe and effective benzodiazepine-free option for preventing and/or treating alcohol withdrawal in small burn patients.

